# Heart Repertory

**Published:** 1897-06

**Authors:** 


HEART REPERTORY. By John H. Clarke, M. D., F. R. G. S., F. B. H. S., Ext. Mem. Roy. Med. Soc. Edin., Consulting Physician to the London Homeopathic Hospital. Compiled mainly from the author's work on *Diseases of the Heart and Arteries.* London: E. Gould & Son, 59 Moorgate Street, E. C.

This little volume, as stated on the title page, copied above, is compiled from the author's previous work on the heart and arteries, which was reviewed in THE HOMEOPATHIC PHYSICIAN for November, 1895, page 529.

The little book now under notice is, therefore, a repertory *to* the preceding work. It is divided into chapters as follow: Diseases by Name; Causation; Respiration; Chest; Chest, left side; Chest and Heart; Chest and Side; Chest and Sternum; Sternum; Heart, region of; Heart; Apex of Heart; Base of Heart; Beneath Heart; Left Heart; Right Heart palpitation; Fainting and Faintness; Circulation; Pulse; Aggravations and Ameliorations; Concomitants. It must be included, and make one more, in the list of valuable monograph repertories which every good homeopathic physician has upon his shelves for ready reference.

